# Prognostic model based on centrosome-related genes constructed in head and neck squamous cell carcinoma

**DOI:** 10.7150/jca.102057

**Published:** 2024-10-21

**Authors:** Peng Zhang, Chunrong Xiong, Dunhui Yang, Kang Li, Zhen Wang, Fang Ma, Xianqin Liao, Miao Xie, Xianhai Zeng

**Affiliations:** 1 Department of Otolaryngology, Longgang Otolaryngology Hospital & Shenzhen Key Laboratory of Otolaryngology, Shenzhen Institute of Otolaryngology, Shenzhen, Guangdong, China.; 2 School of Computer Science and Engineering, Yulin Normal University, Yulin, 537000, China.; 3 Department of Graduate and Scientific Research, Zunyi Medical University, Zunyi, 563000, China.

**Keywords:** centrosome, prognostic model, HNSCC, immune infiltration

## Abstract

Head and neck squamous cell carcinoma (HNSCC) is the most common malignant tumor in the epithelium of the head and neck. The role of the centrosome in malignant tumors is crucial. However, research on the centrosome in HNSCC remains largely unexplored. In this study, bioinformatics tools were utilized to analyze the expression and prognostic significance of centrosome-related genes (CRGs). CRGs exhibited a relatively high mutation frequency in HNSCC. Consensus unsupervised clustering analysis based on the expression profiles of CRGs revealed significant associations with clinical features, prognosis and immune microenvironment in HNSCC. Prognostic features were constructed using univariate and LASSO Cox regression, resulting in a centrosome-related model with eleven features. Patients were classified into high-risk and low-risk groups based on median risk scores. External validation using the GSE41613 dataset from the GEO database confirmed the reliability of the centrosome-related model. The model was associated with the prognosis of HNSCC patients, and centrosome-related features could impact tumor prognosis by influencing the tumor immune microenvironment. Finally, qPCR showed that CRGs were highly expressed in tumor tissues. This study developed a novel centrosome-related prognostic model, applicable for predicting the prognosis and immune landscape of HNSCC patients, offering potential targets for future HNSCC treatment.

## Introduction

Head and neck squamous cell carcinoma (HNSCC) originates from the oral cavity, oropharynx, larynx, and hypopharynx, representing a highly aggressive malignant tumor with poor prognosis^1^. Squamous cell carcinoma is the most common cancer type in the head and neck region and ranks as the sixth most prevalent cancer globally^2^. Major risk factors associated with HNSCC include, but are not limited to, extensive tobacco and alcohol use, HPV or EBV infections, and genetic factors^3^. Some of these risk factors exhibit geographical variations; for instance, betel nut chewing is most prevalent in India, while exposure to carcinogenic air pollutants is more common in developing regions like India and China^4^. The incidence of HPV-driven HNSCC is increasing in Western countries^5^, while EB virus-driven HNSCC is more common in East Asian developing countries^6^. HNSCC is characterized by a high rate of cervical lymph node metastasis, increased invasiveness and recurrence, leading to poor prognosis. Currently, the primary treatments for HNSCC include surgery, radiotherapy, and chemotherapy^7^, with targeted therapy and immunotherapy emerging as novel cancer therapies. Unfortunately, the overall response rate to these treatments is unsatisfactory. Therefore, it is crucial to identify satisfactory biomarkers or methods that can accurately predict the survival of HNSCC patients to improve their prognosis. Thus, there is an urgent need to identify prognostic features and potential mechanisms of HNSCC development.

The centrosome is the smallest organelle in eukaryotic cells, serving as the microtubule organizing center and a key regulator of cell division^8^. The centrosome consists of two barrel-shaped centrioles and surrounding pericentriolar material (PCM)^9^. The centrioles have a diameter of 0.16-0.23 μm and a length of 0.16-0.56 μm, arranged perpendicularly in pairs^10^. The centrioles are surrounded by a series of proteins, and the PCM is essential for centrosome replication as it mediates the formation and stability of daughter centrioles^11^. Each component of the centrosome has its own function. The centrosome has complex biological functions, mainly relying on its structure and a large number of centrosome-related proteins. The centrosome acts as a coordinating center in eukaryotic cells, regulating signal transduction, cell division, polarity, and migration^12^. The structure, function, and number of centrosomes are strictly controlled within cells. Similar to DNA, the centrosome replicates once per cell cycle in a semi-conservative manner^13^, ^14^. To form an effective bipolar mitotic spindle, the centrosome must replicate during the S phase, and centrosomes mature and separate during the G2 phase^15^. Finally, each daughter cell inherits one centrosome after mitosis. Any disruption of its function may lead to spindle disorganization and chromosome missegregation, ultimately resulting in chromosomal instability (CIN) and aneuploidy. Distortions in centrosome size, shape, number, and position are collectively referred to as centrosome amplification (CA)^16^. Centrosomal abnormalities are common in malignant tumors, and anomalies in centrosome number and structure are observed in almost all human cancers^17^. Furthermore, the degree of centrosomal distortion is correlated with the progression of malignant tumors. Some studies suggest that CA promotes the development of HNSCC, and centrosome overamplification is highly prevalent in HNSCC. Centrosome overamplification serves as a phenotypic marker for HNSCC and can reflect various genotypic changes^18^. Researchers have been delving into the role of the centrosome in cancer development and progression. However, to date, there is no relevant research on a centrosome-related prognostic model in HNSCC. Therefore, investigating centrosome-related gene markers in HNSCC may provide valuable therapeutic guidance in clinical practice.

## Materials and Methods

### Collection and Preprocessing of HNSCC

Training Set: We selected 521 patients from The Cancer Genome Atlas (TCGA) database and downloaded their mRNA expression profiles and clinical information from the Genomic Data Commons (GDC) (https://portal.gdc.cancer.gov/). The expression matrix included exon model fragments per kilobase of exon model per million mapped fragments (FPKM) and count values. After removing cases with missing follow-up clinical information, the remaining HNSCC and normal patients were included in our training cohort.

We downloaded somatic mutation data from the Genomic Data Commons (GDC, https://portal.gdc.cancer.gov/) in mutation annotation format (MAF). The mutation data, sorted in MAF format, was analyzed, and the Tumor Mutational Burden (TMB) was calculated using the "maftools" R package^19^.

Validation Set: We obtained an external dataset GSE41613 from the Gene Expression Omnibus (GEO) database (https://www.ncbi.nlm.nih.gov/geo/) containing 97 samples. After excluding cases with death due to other factors, 76 samples were retained and utilized as our test set.

### Selection of Differentially Expressed CRGs

We collected a total of 727 CRGs from public databases and literature. Subsequently, using the "limma" package and setting the threshold of p < 0.05 20, we identified 601 differentially expressed CRGs (DEGs) in normal and HNSCC tissues from TCGA. Additionally, a heatmap was generated to visually display the significant expression differences of these DEGs between normal and HNSCC tissues.

### Protein-Protein Interaction (PPI) Analysis

Functional interactions among proteins are crucial for understanding the molecular mechanisms of cancer. The Search Tool for the Retrieval of Interacting Genes (STRING v11.0) online tool (https://string-db.org/) was employed to establish potential interactions among a large number of genes^21^. Overlapping DEGs were input into the software to construct a PPI network, visualized using Cytoscape software 3.7 (http://www.cytoscape.org)^22^.

### Consensus Clustering and Evaluation of Expression, Prognosis, and Immune Infiltration

Consensus clustering analysis was performed using the "ConsensusClusterPlus" package in R with the optimal k value determined from 100 iterations on 80% of the total samples^23^. The "ConsensusClusterPlus" package provided a clustering heatmap displaying the optimal k value. Through heatmap analysis, we differentiated the expression levels of CRGs between clusters 1 and 2. The "survival" package in R was used to assess overall survival between clusters 1 and 2. The "limma" R package was employed for differential expression gene (DEGs) analysis using raw counts. The volcano plot was used to display DEGs with adjusted p-value < 0.05 and |log2 fold change| > 1.

### Functional Enrichment Analysis of Differentially Expressed Genes and Tumor Mutational Burden (TMB)

The "clusterProfiler" R package was used to perform Gene Ontology (GO) and Kyoto Encyclopedia of Genes and Genomes (KEGG) pathway enrichment analysis^24^, ^25^. Indicators with a false discovery rate (FDR)-adjusted p-value less than 0.05 using Fisher's exact test were considered significant. Gene Set Enrichment Analysis (GSEA) was conducted using the "clusterProfiler" package with gene set files c2.cp.kegg.symbols.gmt and c5.go.symbols.gmt. To compare mutation profiles between high/low expression groups, we assessed Tumor Mutational Burden (TMB) levels, the optimal quantitative standard reflecting mutation levels. TMB scores were calculated using the "tmb" function in the "maftools" package.

### Evaluation of Immune Microenvironment in HNSCC

We utilized the "estimate" package to calculate stromal, immune, and ESTIMATEScore, as well as tumor purity from HNSCC samples from TCGA. Subsequently, we used CIBERSORT to estimate the infiltration levels of several tumor-infiltrating immune cells in the tumor immune microenvironment (TIME)^26^. Additionally, we analyzed the expression of leukocyte antigen genes in high/low expression subgroups and described the tumor immune microenvironment further by examining the expression of 11 immune checkpoint genes and evaluating immune response scores using the "ssGSEA" algorithm.

### Construction and Validation of the Centrosome-Related Prognostic Model

The TCGA validation cohort and an external cohort (GSE41613 dataset) were further used to validate the prognostic efficacy of centrosome-related gene features. Consequently, single-factor Cox analysis of overall survival (OS) was employed to screen CRGs with prognostic value. Subsequently, LASSO regression with 10-fold cross-validation was executed with 1,000 cycles using the "glmnet" R package, and 1,000 random stimuli were set. Based on the optimal λ value, the optimal genes were selected to build the model, termed the Centrosome-Related Survival Score (CRSS). The CRSS was calculated based on the expression levels of each gene and its corresponding regression coefficient using the following formula:



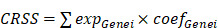



Patients were then categorized into high-risk and low-risk groups based on the optimal cutoff value, determined using the "survminer" R package. The predictive sensitivity of CRSS was visualized using the "survivalROC" R package. The model's effectiveness was assessed in the validation set using the same coefficients and cutoff values as the training set.

### Construction of Nomogram and Calibration Plot

In this study, a Cox regression model and the "rms" package in R were used to construct an operating system prediction nomogram, setting 1-year, 3-year, and 5-year OS as endpoints. A calibration plot was employed to visualize the consistency between predicted 1-year, 3-year, and 5-year OS and actual OS.

### Univariate and Multivariate Cox Regression

Univariate Cox regression was performed on TCGA-HNSCC, including gene expression and overall survival. Multivariate Cox regression was employed to assess independent risk factors in the same cohort. Genes and factors with a false discovery rate (FDR) < 0.05 were considered statistically significant. Results of univariate and multivariate Cox regression were obtained and visualized using the "ggforest" function in the "survminer" package. Clinical Correlation Analysis of High/Low Risk Subgroups in the Centrosome-Related Prognostic Model was performed. The clinical correlation heatmap of high/low-risk subgroups in the Centrosome-Related Prognostic Model.

### Evaluation of the Immune Microenvironment in HNSCC

We used the "estimate" algorithm to calculate stromal, immune, and ESTIMATEScores, as well as tumor purity, from breast cancer samples from TCGA. Next, we employed algorithms including CIBERSORT to estimate the levels of immune cell infiltration in the tumor immune microenvironment (TIME). We further analyzed the correlation between risk scores and immune cells, validated immune functional differences between high and low immune function groups, and performed Kaplan-Meier analysis on the survival curves of patients with high and low immune function.

### Prediction of Immunotherapy Sensitivity and Drug Response

To validate the predictive value of immunotherapy response, an additional immunotherapy dataset was downloaded from http://tide.dfci.harvard.edu/. This dataset was used to predict immunotherapy responses. To identify variations in mutated genes between high and low expression groups of CRGs, mutation analysis was conducted using the "TCGAmutations" and "maftools" R packages. The tumor mutational burden (TMB) of TCGA samples was calculated using the "tmb" function in the "maftools" package. Furthermore, we analyzed the correlation between high/low-risk groups and immune cells. We downloaded the Head and Neck Squamous Cell Carcinoma (HNSCC) TIDE dataset, which provides information on tumor immune dysfunction and exclusion data^27^. The TIDE algorithm accurately predicts the efficacy of immunotherapeutic drugs based on TIDE scores.

### RNA Extraction and Real-time Quantitative PCR (qPCR)

Total RNA was extracted from cells and tissues using Trizol reagent (Invitrogen, USA) according to the manufacturer's instructions. Subsequently, reverse transcription of RNA into cDNA was performed using the PrimeScript RT reagent kit and gDNA Eraser (Takara, Japan). SYBR Green dye qPCR analysis was carried out on the cDNA. Primer sequences were listed in Supplementary Table S1.

### Statistical Analysis

The Wilcoxon rank-sum test was employed for differential analysis using the `wilcox.test()` function to calculate the p-value of the Wilcoxon rank-sum test for each gene between the normal and tumor groups. Single-variable Cox analysis was conducted on overall survival (OS) to determine relevant genes and their prognostic value. Kaplan-Meier survival curves were generated, and the log-rank test was used to compare between the two groups. Spearman correlation analysis was used to assess the correlation between the risk score of the prognostic model and the immune score. All statistical analyses were conducted using R version 4.1.1 (https://www.r-project.org/) and appropriate packages. Statistical significance was set at p < 0.05.

## Results

### Identification and Protein-Protein Interaction (PPI) Network of Differentially Expressed CRGs in HNSCC

**Figure 1:**
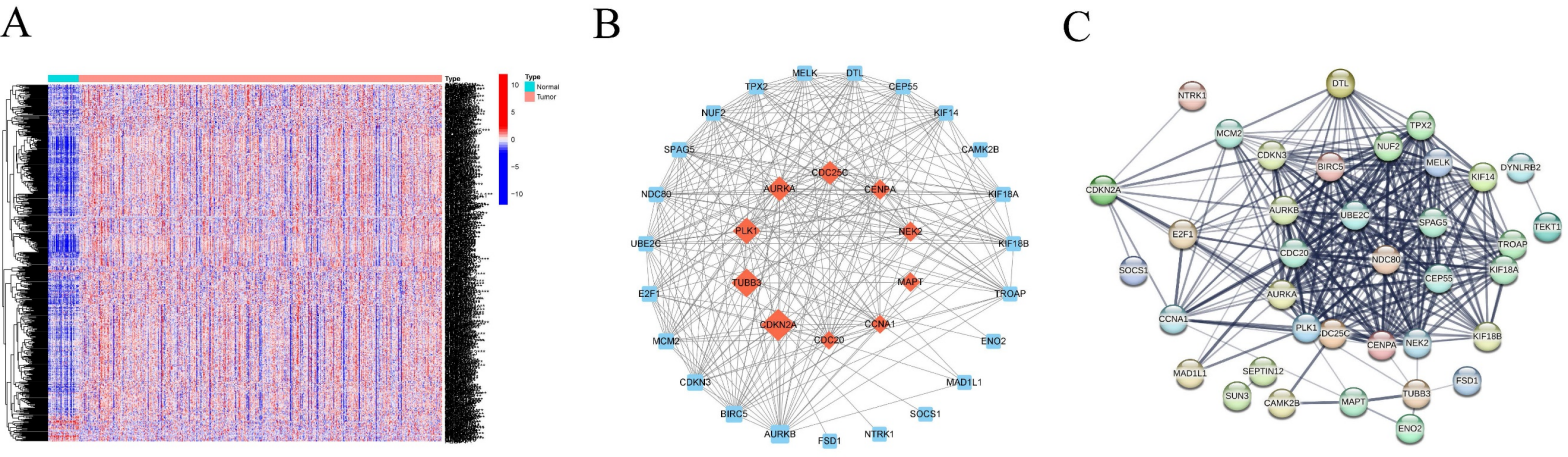
(A)Heatmap of centrosome-associated DEGs by comparing the top 50 up-regulated and top 50 down-regulated centrosomes in HNSCC tissues in TCGA with normal tissues. (B-C) PPI interaction network of centrosome-associated DEGs (|logFC| = 2.0, p < 0.05).

By consulting public databases and literature, we collected a total of 727 CRGs and validated the expression of 699 genes in our training set. Subsequently, we employed the "DESeq155" algorithm with the "limma" test to identify 601 centrosome-related Differentially Expressed Genes (DEGs) between normal and HNSCC samples from TCGA. A heatmap further illustrated the expression patterns of these DEGs in normal and HNSCC tissues (Figure 1A). The 47 differentially expressed genes were uploaded to the STRING database and Cytoscape to construct a PPI network (Figure 1B and 1C).

### Optimal Grouping and Prognostic Evaluation of CRGs

**Figure 2:**
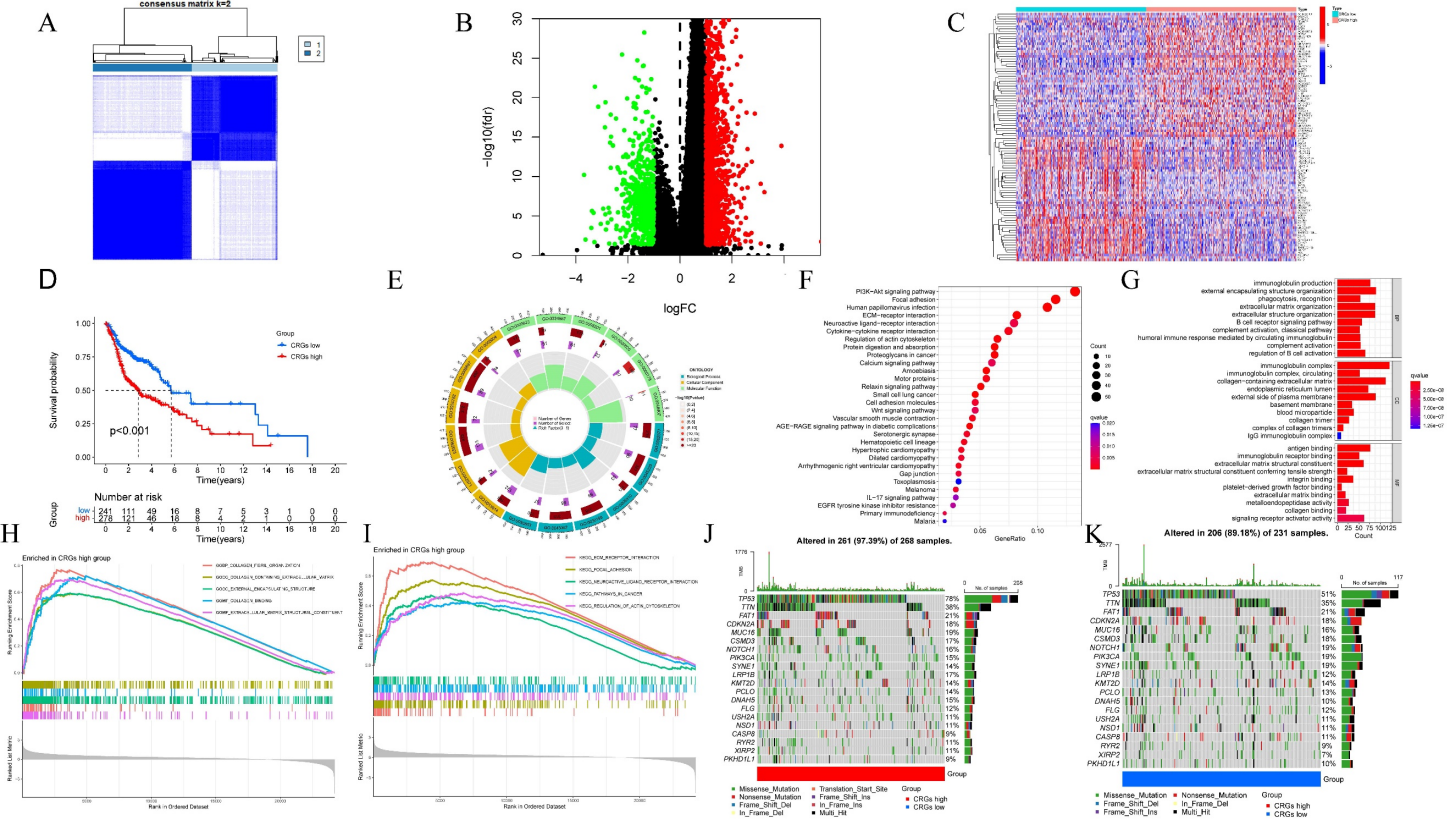
(A) Optimal consensus clustering matrix k=2. (B)Volcano plot of the expression of centrosome-associated DEGs found by comparing the high-expression group with the low-expression group. Green represents down-regulated genes and red represents up-regulated genes. (C)Heat maps displaying the top 50 upregulated and top 50 downregulated centrosome-associated DEGs between high-expression group and the low-expression group. (D) Kaplan-Meier analysis of OS curves of patients in the TCGA high/low expression group. (E-F) GO enrichment of centrosome-associated DEGs. (G) KEGG pathways upregulated by centrosome-associated DEGs. (H-I) Genomic enrichment analysis (GSEA) of high/low expression groups of centrosome-related genes (J-K). Mutant genes between high/low expression groups. Mutation information of each gene in each sample was shown in the waterfall plot, with various color annotations to distinguish different mutation types. The barplot above the legend exhibited the mutation burden, and the other barplot on the right showed the distribution of mutation types among the top 20 genes.

To understand the value of CRGs, we conducted a consensus clustering analysis. Based on the clustering heatmap, we chose the optimal k value as 2 for subsequent analysis (Figure 2A). After sorting out the data, extract the expression level of each CRGs gene, and divide the samples into multiple high-expression groups and low-expression groups by using the median expression level of each gene. Finally, perform differential analysis on the corresponding high and low groups, and screen out differentially expressed genes from the results according to the set fold change and FDR screening value. A total of 1860 DEGs were identified, including 1244 upregulated and 616 downregulated DEGs (Figure 2B). Heatmap analysis indicated higher expression levels in Group 2 compared to Group 1 in HNSCC (Figure 2C). Furthermore, survival analysis demonstrated that patients in Cluster 1 had a significantly longer survival probability [p < 0.001] than those in Cluster 2 (Figure 2D). These results suggest that consensus clustering provides preliminary stratification of the risk in HNSCC patients.

### Identification and Functional Inference Analysis of HNSCC DEGs

To elucidate the potential mechanisms of CRGs in HNSCC, Gene Ontology (GO) analysis was performed, providing terms for Cellular Component (CC), Molecular Function (MF), and Biological Process (BP). The results indicated enrichment of DEGs in various GO terms, including humoral immune response, processes based on intermediate filaments, and receptor ligand activity (Figure 2E-F). KEGG pathway enrichment analysis revealed enrichment in pathways such as neuroactive ligand-receptor interaction, protein digestion and absorption, and estrogen signaling pathway (Figure 2G). Given the association between centrosome expression levels and tumor grade and prognosis in tongue cancer patients, we proposed the hypothesis that elevated centrosome expression accelerates tumor growth. GSEA analysis was conducted to elucidate the biological functions and pathways associated with the risk score. The results indicated a dynamic correlation between the high expression of CRGs and tumor features such as GOBP COLLAGEN FIBRIL ORGANIZATION, COLLAGEN CONTAINING EXTRACELLULAR MATRIX, EXTRACELLULAR MATRIX STRUCTURAL CONSTITUENT, ECM RECEPTOR INTERACTION, FOCAL ADHESION, NEUROTINVE LIGAND RECEPTOR INTERACTION, RHYTHMYS IN CANCER, and REGULATION OF ACTIN CYTOSKELETON. Conversely, immune-related functions like IMMUNOGLOBULIN COMPLEX, T CELL RECEPTOR COMPLEX, ANTIGEN BINDING, IMMUNOGLOBULIN RECEPTOR BINDING, ALLOGRAFT REJECTION, ARACHIDONIC ACID METABOLISM, IMMUNE NETWORK FOR IGA PRODUCTION, OLEIC ACID METABOLISM, and PRIMARY IMMUNODEFICIENCY were significantly enriched in the low expression group (Figure 2H-I; Figure S1). To explore the independent prognostic value of CRGs, we examined the genomic mutations of these genes in HNSCC. Tumor Mutational Burden (TMB) analysis showed that TP53, TTN, and FAT1 had high mutation frequencies in both high and low expression groups, with a higher mutation frequency in the high expression group (Figure 2J-K). Kaplan-Meier analysis of progression free survival curves for TCGA patients in the DRGs high/low groups showed that the high-expression group had a shorter survival time in most cases (Figure S2).

### Association of CRGs with the Tumor Immune Microenvironment (TIME) in HNSCC

**Figure 3:**
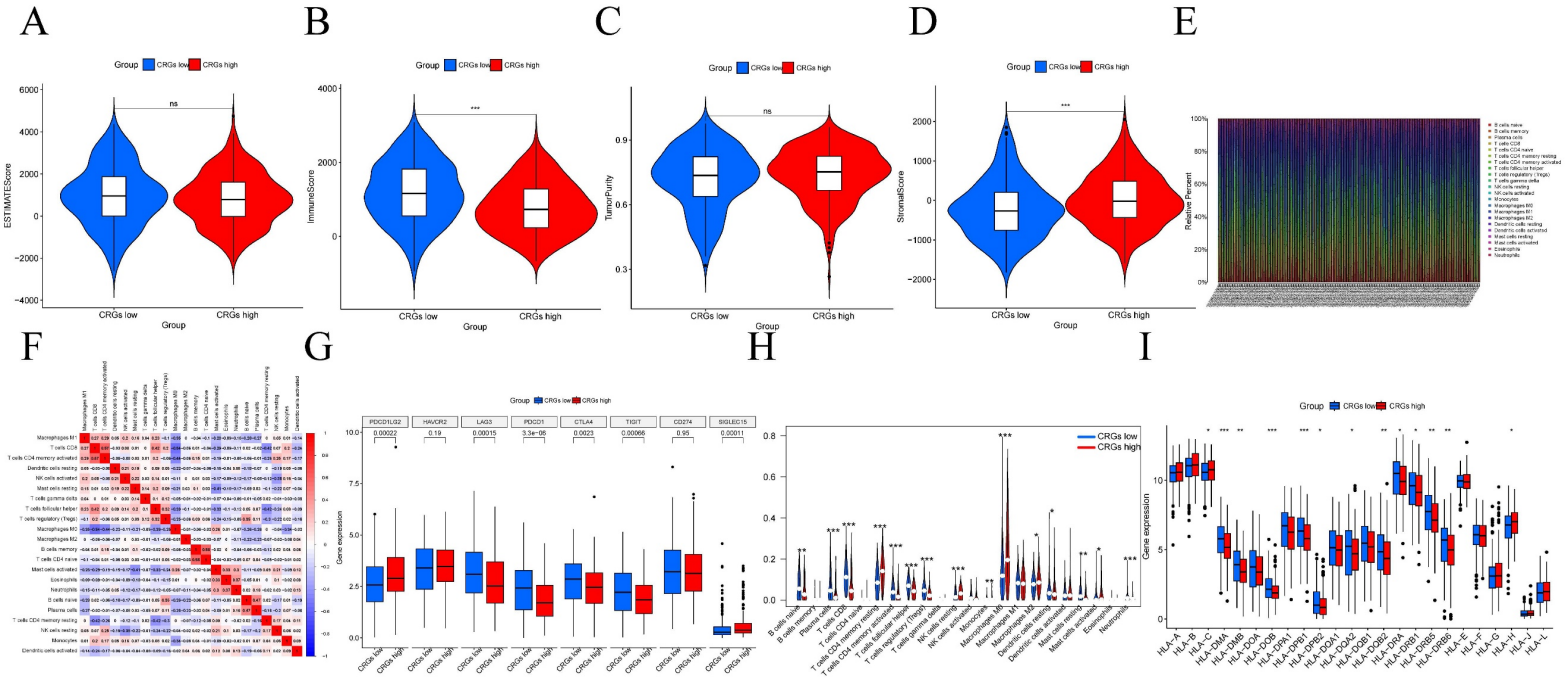
Diversity of the tumor immune microenvironment in patients with high/low expression (A) ESTIMATE scoring of the high/low expression subgroup (B) Immunity scoring of the high/low expression subgroup. (C) Stromal cell scoring of the high/low expression subgroup. (D) Tumor purity in the high/low expression subgroup. (E)The percentage of each type of immune cell in high/low expression subgroup. (F) Heatmap showing the correlation between 22 kinds of TICs and numeric in each tiny box indicating the p value of correlation between two kinds of cells. The shade of each tiny color box represented corresponding correlation value between two cells, and Pearson coefficient was used for significance test. (G)Relationship between high/low expression subgroups and immune checkpoints. (H) Violin plot visualize 22 immune cell infiltrations between high/low expression subgroups. (I) Box plots show the differences in expression of HLA family genes between high/low expression subgroups.

We further investigated the Tumor Immune Microenvironment (TIME), which plays a crucial role in tumor development and treatment response. We assessed stromal scores, immune scores, and tumor purity in different risk groups using the ESTIMATE and CIBERSORT algorithms. ESTIMATE is a method that evaluates the matrix-immune comprehensive score, matrix content score, immune cell infiltration level, and tumor purity of each HNSCC sample (Figure 3A-D). The CIBERSORT method was applied to study the patterns of immune cells. This was followed by elucidation of the composition of immune cells in HNSCC samples and their relationships (Figure 3E-F). We then further validated the relationship between high/low risk subgroups and immunotherapy and immune cell infiltration. The expression of immune checkpoint genes differed between high/low expression subgroups (Figure 3G). Notably, we observed that CRGs displayed dysregulated levels in various immune cells, including regulatory T cells (Tregs), follicular helper T cells, CD4 memory-activated T cells, CD8 T cells, M0 macrophages, activated dendritic cells, quiescent mast cells, and eosinophils (Figure 3H)^28^. Additionally, HLA genes are closely related to tumor immunity. We examined whether the expression of HLA-related genes differed in the risk subgroups. The expression of HLA genes in high-risk individuals was generally lower than that in low-risk individuals (Figure 3I).

### Construction of the Centrosome-Related Prognostic Model for HNSCC

**Figure 4:**
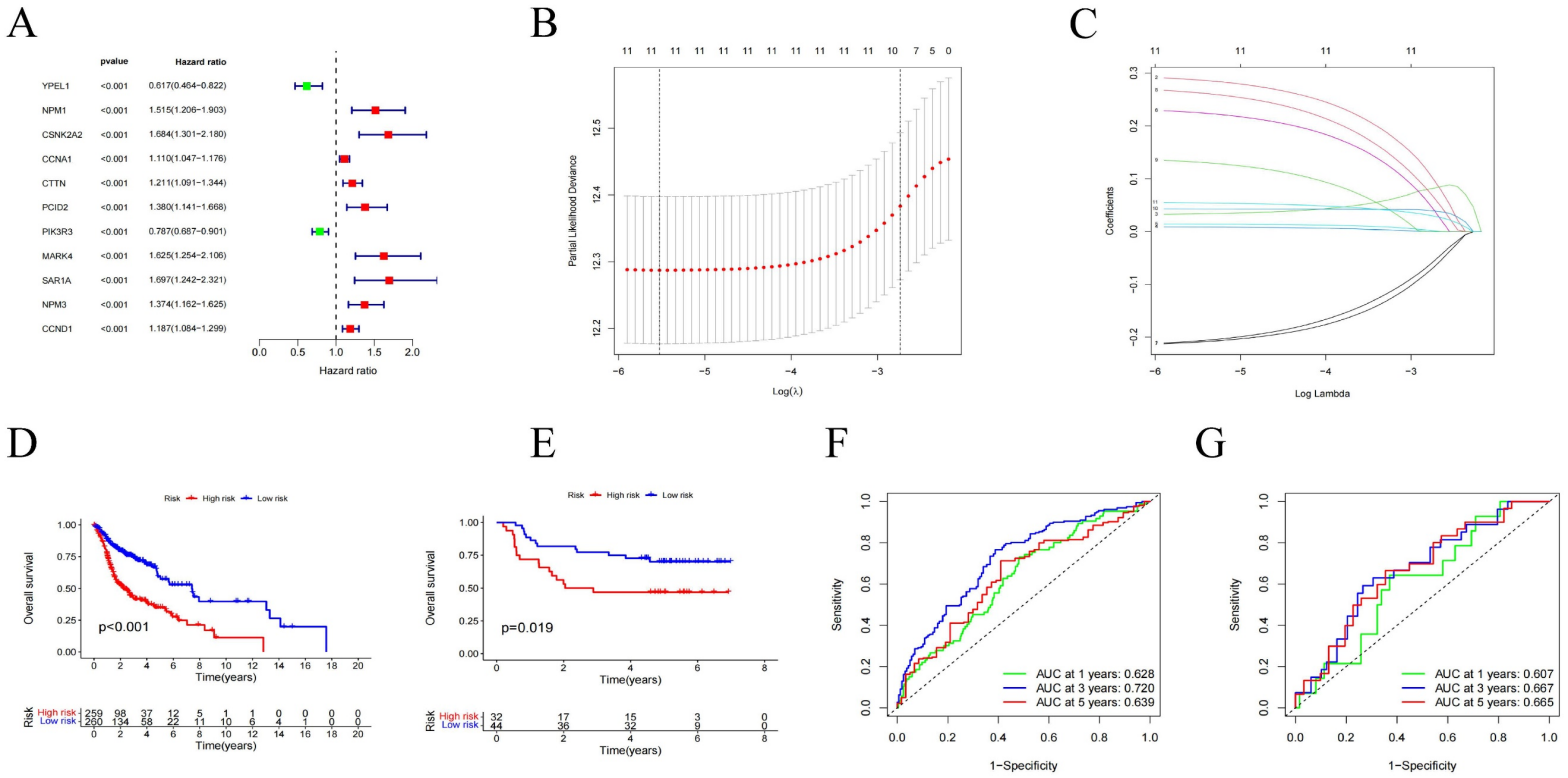
Centromere-related prognostic modeling and stability validation. (A) Univariate Cox regression analysis of eleven genes based on cross-validation and least partial likelihood bias to further demonstrate independent prognostic-related genes and obtain gene indices. (B-C) Least Absolute Shrinkage and Selection Operator (LASSO) Cox regression for centromere-associated prognostic genes. (D) Kaplan-Meier analysis of OS curves for TCGA patients in the high/low risk subgroup of the training cohort. (E) Kaplan-Meier analysis of OS curves for GEO patients in the high/low risk subgroup of the validation cohort. (F) Time-dependent ROC analysis showing the prognostic value of centroid-related prognostic models in the training set. (G) Time-dependent ROC analysis showing the prognostic value of centrosome-related prognostic models in the validation set.

Using univariate Cox regression analysis, we selected 11 genes significantly associated with the prognosis of HNSCC patients (p < 0.05) from centrosome-related DEGs. These genes included YPEL1, NPM1, CSNK2A2, CCNA1, CTTN, PCID2, PIK3R3, MARK4, SAR1, NMP3, and CCND1 (Figure 4A). To avoid overfitting and bias, we employed LASSO regression analysis on the results of univariate regression analysis using the "glmnet" R package. Through cross-validation, we established a prognostic risk model with 11 genes as follows: Risk Score = [YPEL1 expression × (-0.20744)] + [NPM1 expression × (0.28728)] + [CSNK2A2 expression × (0.03336)] + [CCNA1 expression × (0.00828)] + [CTTN expression × (0.91382)] + [PCID2 expression × (0.22536)] + [PIK3R3 expression × (-0.20961)] + [MARK4 expression × (0.26322)] + [SAR1A expression × (0.13187)] + [NPM3 expression × (0.94290)] + [CCND1 expression × (0.05443)] (Figure 4B-C).

### Construction and Validation of the Centrosome-Related Prognostic Model

**Figure 5:**
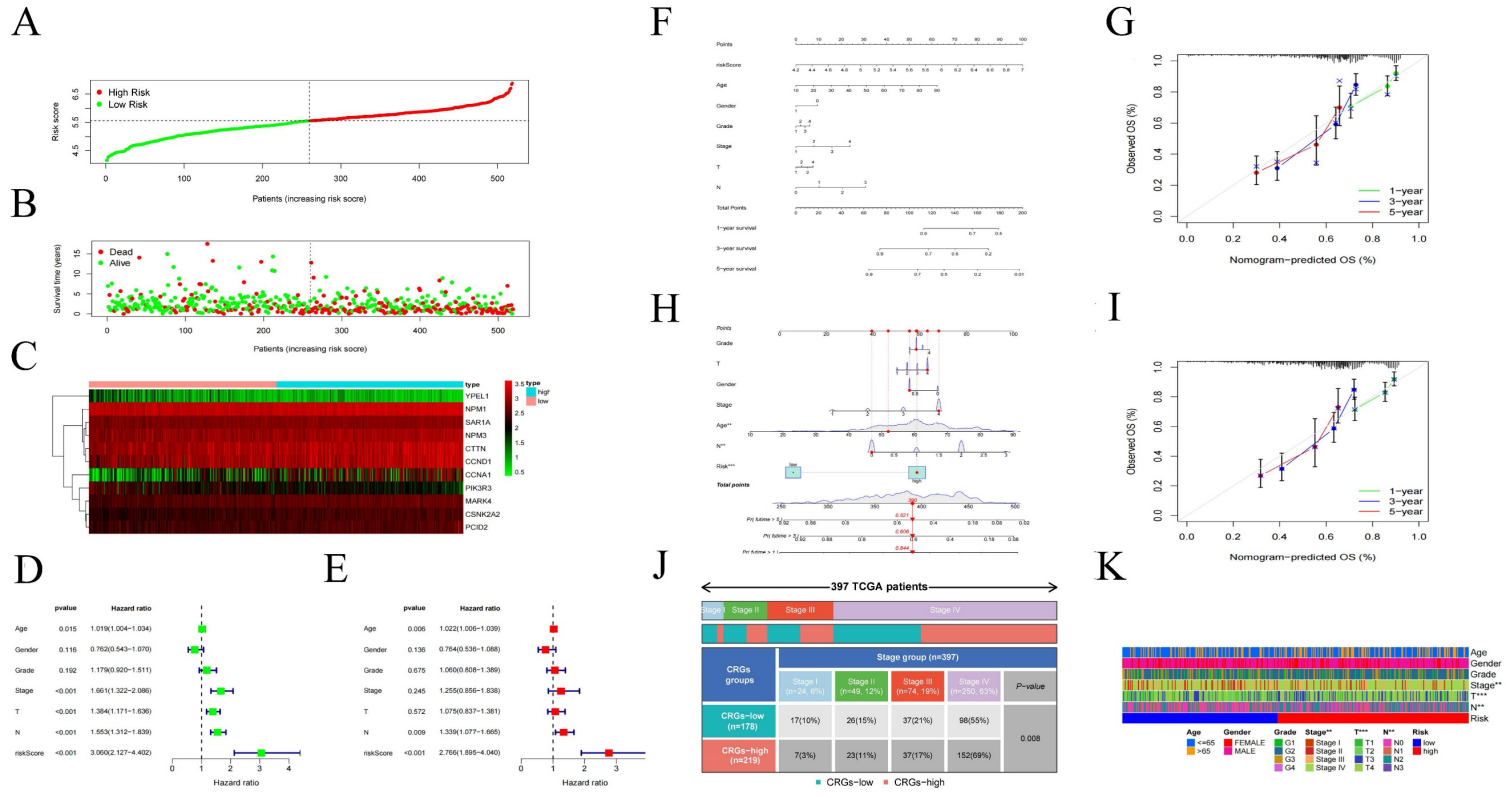
(A-C) Distribution of the training set and patterns of survival time and survival status between high/low risk subgroups. (D-E) One-way Cox analysis and multifactor Cox regression analysis of centrosome prognostic models. (F-I) Nomogram integrating risk score and clinical features based on the TCGA training dataset and validation dataset. Nomogram integrating risk score for predicting the OS rates at 1-, 3-, and 5-year of HNSCC patients. Calibration curve to evaluate the ability of Nomogram to predict OS at 1-, 3-, and 5-year HNSCC patients. (J) Clinical correlation analysis of the centrosome high/low risk subgroup prognostic model. (K) Heatmap of clinical correlations for the centrosome high/low risk subgroup prognostic model. Asterisks represent statistical p values (*p < 0.05; **p < 0.01; ***p < .001).

After grouping, prognosis analysis revealed significantly worse outcomes for the high-risk group. Patients in the high-risk group had significantly shorter overall survival than those in the low-risk group (Figure 4D, p < 0.001). To further validate the robustness of this feature's prognostic value, we calculated the area under the curve (AUC) for predicting 1-year, 3-year, and 5-year overall survival (OS), resulting in AUC values of 0.628, 0.720, and 0.639, respectively (Figure 4F). Subsequently, we sought to validate the prognostic prediction ability of the model using an independent dataset. After excluding cases with duplicated or incomplete survival information and correcting batch effects, we used the GSE41613 dataset as a validation cohort. Consistent with the results from the training cohort, patients in the high-risk group in the validation cohort exhibited shorter survival times than those in the low-risk group (Figure 4E). The 1-year, 3-year, and 5-year ROC curves based on both the training and validation cohorts are shown demonstrating satisfactory prognostic value (Figure 4G). Dot plots indicated lower overall survival rates for patients with higher risk scores in each dataset. Additionally, differences in the expression of the 11 prognostic CRGs were observed between the high-risk and low-risk groups (Figure 5A-C). These results suggest that the centrosome-related prognostic model based on these eleven candidate genes exhibits high accuracy and stability in predicting HNSCC prognosis.

To evaluate the accuracy of the model, we performed univariate and multivariate Cox regression analyses, incorporating pathological features. Univariate Cox regression analysis revealed associations between age, N stage, risk score, and the prognosis of HNSCC patients (Figure 5D-E). Similarly, multivariate Cox regression analysis showed associations between age, stage, T stage, N stage, and risk score with the prognosis of HNSCC patients (Figure 5F-I). Additionally, we constructed nomograms based on risk score, age, gender, T stage, N stage and identified that the model had good predictive ability (Figure 5J). Finally, we used a heatmap to demonstrate the relationship between the high/low-risk subgroups of the centrosome-related model and several clinical variables (Figure 5K).

### Association of Centrosome-Related Gene Features with the HNSCC Tumor Immune Microenvironment (TIME)

**Figure 6:**
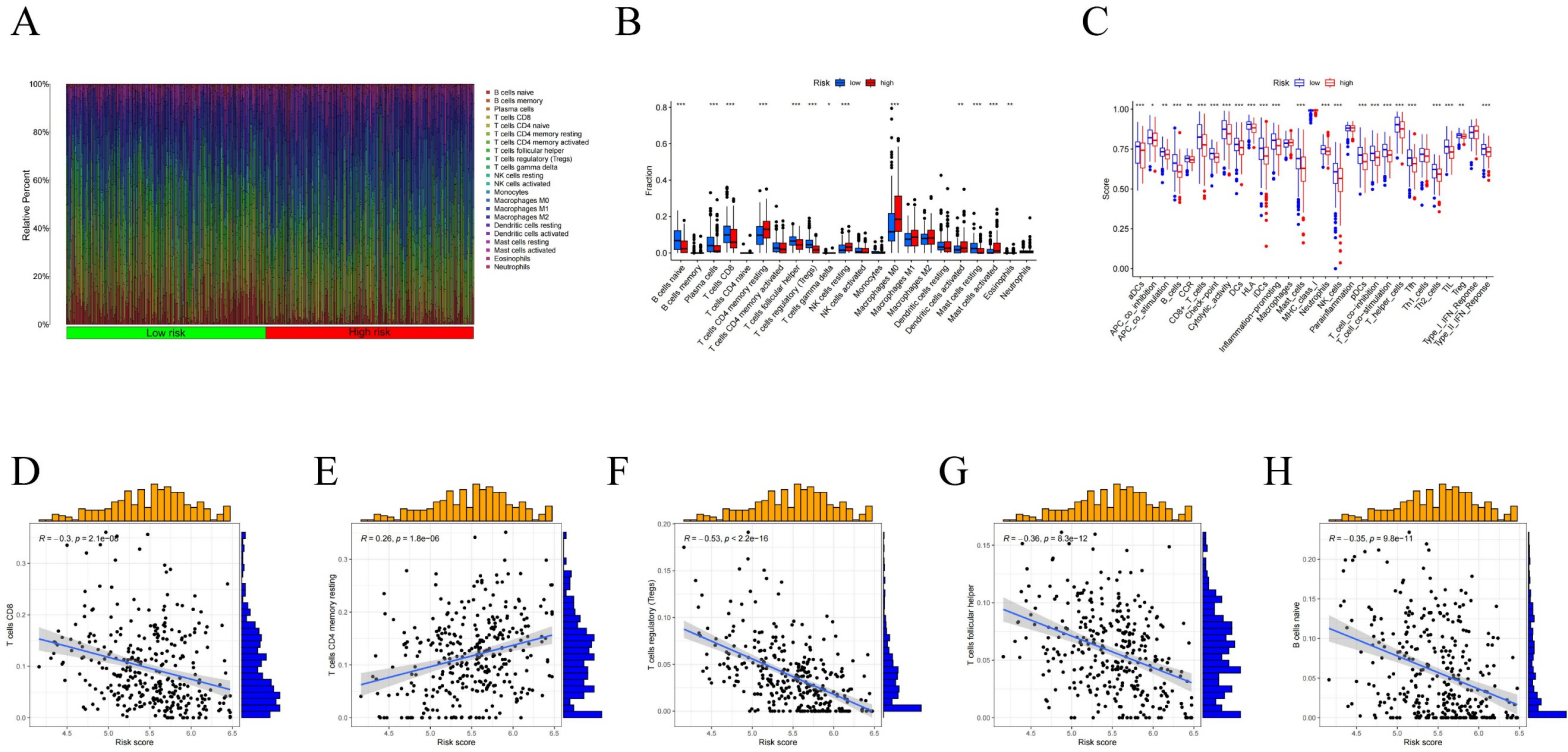
(A) Proportion of immune cell composition in the risk group as determined by the CIBERSORT algorithm. (B) Immune cell infiltration in the low/high-risk groups. Low-risk and high-risk groups are indicated by blue and red box plots. (C) Differences in immune function between high/low risk groups. (D-H) Scatter plots showing the correlation between the risk score and the proportion of T cells CD4, B cells, T cells CD8, T cells follicular helper, T cells regulatory.

**Figure 7:**
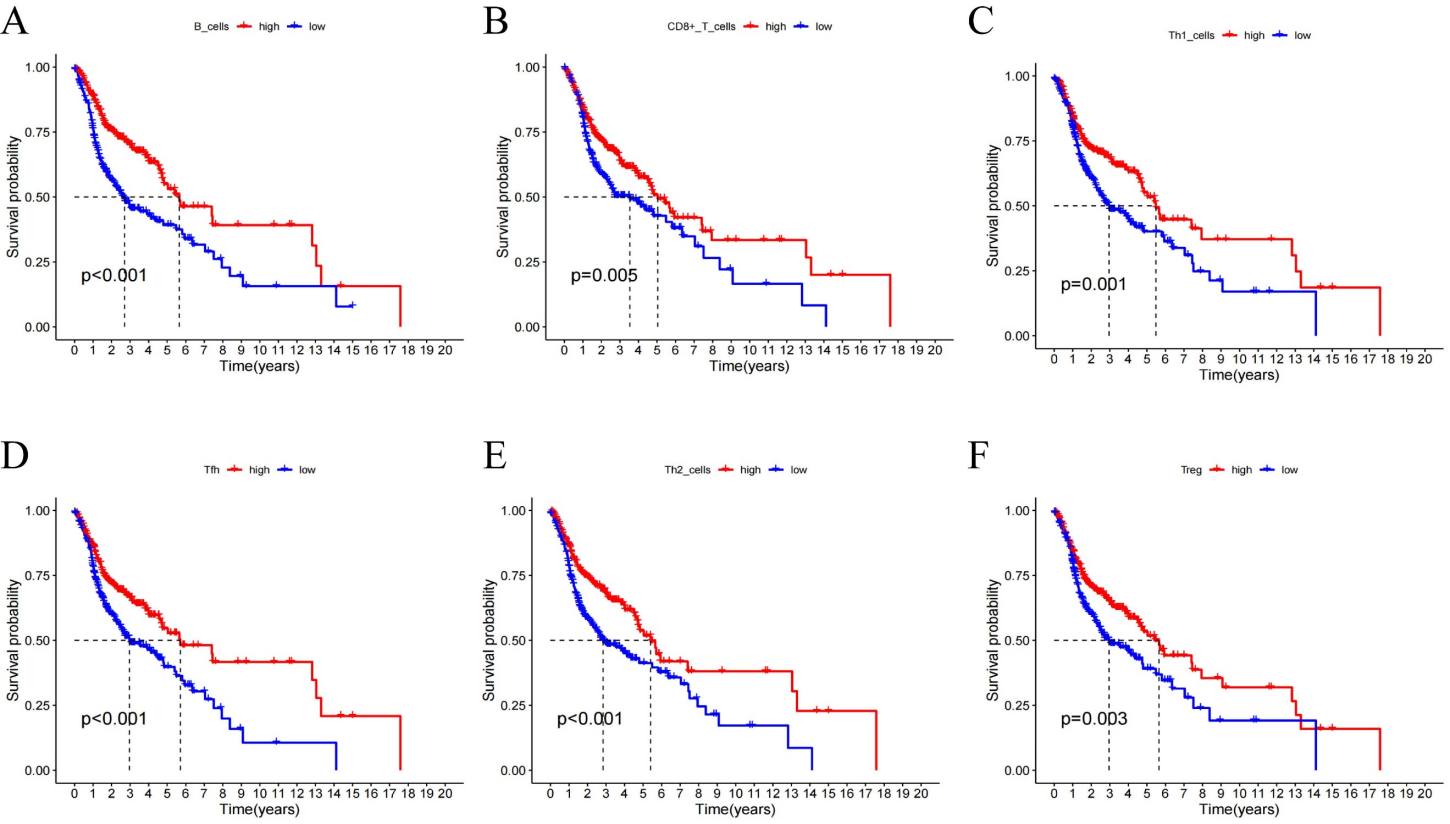
(A-F) Kaplan-Meier analysis was performed on the OS curves of patients with high/low infiltration differences in immune cells. The Kaplan-Meier and log-rank tests for immune cells passed the Wilcoxon rank sum test. Red and blue curves represent high infiltration and low infiltration.

Using the CIBERSORT algorithm, we determined the proportions of immune cell composition in two risk groups of HNSCC (Figure 6A). In this study, we also focused on the tumor-infiltrating cells between subgroups. We found differences in the infiltration of B cells native, plasma cells, T cells CD8, T cells follicular helper, T cells CD4 memory resting, T cells regulatory (Tregs), T cells gamma delta, NK cells resting, Macrophages M0, Dendritic cells activated, Mast cells resting, Mast cells activated, and Eosinophils among different groups (Figure 6B). The ssGSEA analysis showed a significant elevation of most immunocompetent immune cells in the low-risk group between the high-risk and low-risk groups (Figure 6C). By evaluating the risk scores, we found positive correlations of T cells CD4 memory resting with the risk score; while B cells, T cells CD8, T cells follicular helper, T cells regulatory showed negative correlations (Figure 6D-H). Kaplan-Meier analysis of OS curves for patients with diverse immune functions in the high/low-risk groups showed that the high-expression group had a longer survival time in most cases (Figure 7; Figure S3).

### Diverse Treatment Potential in High/Low-Risk Groups

**Figure 8:**
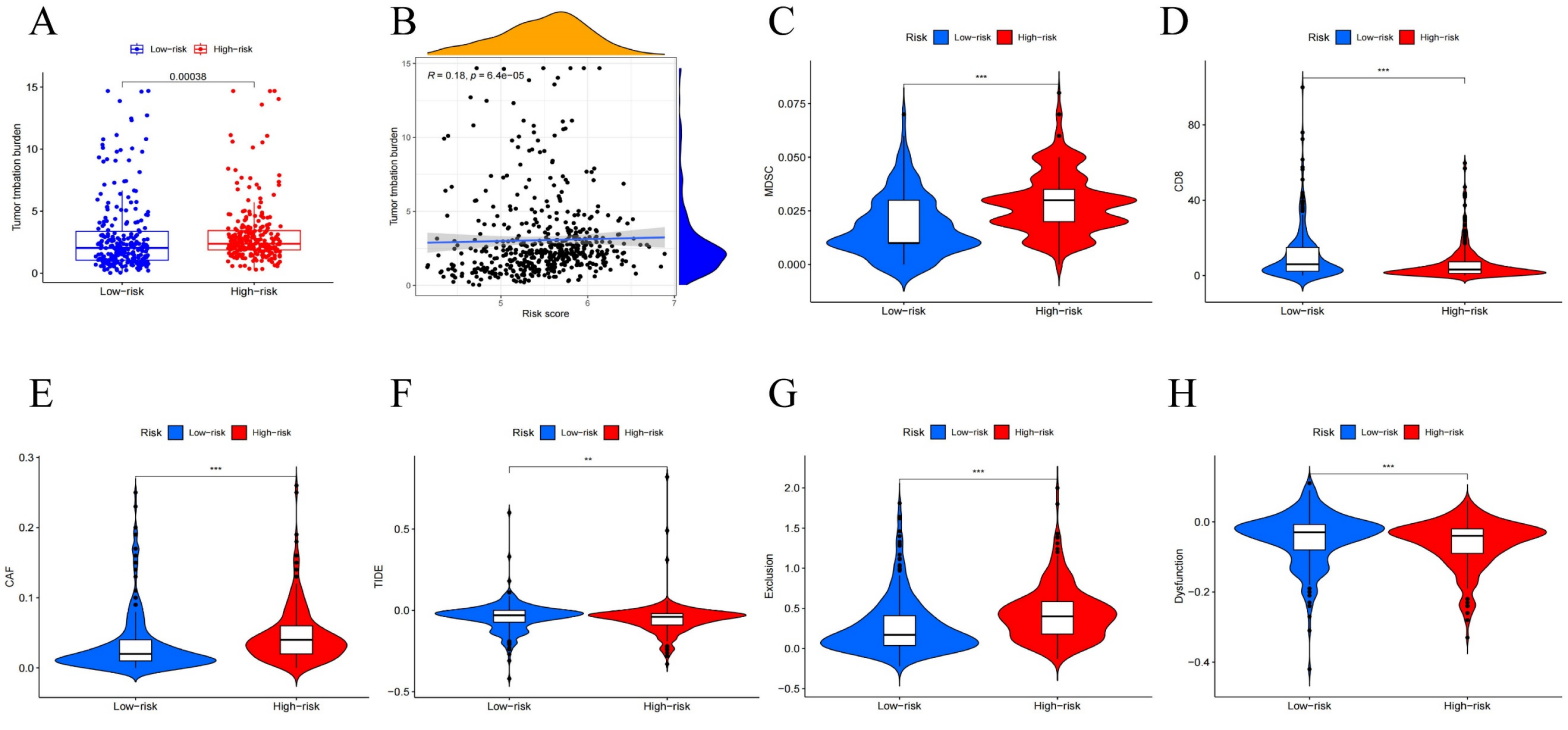
Mutation analysis of centrosome-associated prognostic models. (A) Differences in tumor mutational load (TMB) between high/low risk groups. (B) Correlation between head and neck squamous cancer patients stratified according to TMB values and risk scores. Immune characteristics of different risk subgroups. (C-E) Correlation analysis between high and low risk groups and immune cells. (F-H) Immunotherapy efficacy outcomes, including TIDE, exclusion, and dysfunction scores, between centrosome high/low risk subgroup.

Tumor Mutational Burden (TMB) is generally considered high when mutations exceed 10 or 16 per million base pairs of DNA and is an important factor in tumor development, predicting the efficacy of immune checkpoint blockade and serving as a biomarker for patients benefiting from immunotherapy^29^. To further investigate how the risk-prognostic model predicts tumor development, we studied its relationship with TMB. We calculated TMB scores for patients and found higher TMB scores in the high-risk group (Figure 8A). Correlation analysis between TMB values and risk scores stratified head and neck squamous cell carcinoma (HNSCC) patients, revealing a positive correlation (Figure 8B). Recently, multiple studies have indicated that TMB is closely related to tumor immune cell infiltration and affects the efficacy of immunotherapy. Further analysis revealed higher expression of CAF and MDSC in the high-risk group, while CD8 cells showed significantly higher expression in the low-risk group (Figure 8C-E). Based on the risk features of the centrosome-related gene subtypes, we also compared tumor immune dysfunction and exclusion (TIDE). We further investigated the differences in sensitivity to immunotherapy between patients in the high-risk and low-risk groups using the TIDE prediction score (http://tide.dfci.harvard.edu/). The TIDE algorithm is a recently developed tool for determining the efficacy of tumor immune checkpoint therapy. In this study, we found that the TIDE score in the low-risk group was higher than in the high-risk group. Higher TIDE scores indicate a higher likelihood of immune escape and poorer clinical efficacy of immunotherapy. To further study factors affecting prognosis, we also conducted exclusion and dysfunction scores. We found that the rejection score was significantly higher in the high-risk group (p < 0.001), while the dysfunction score was higher in the low-expression group (p < 0.001) (Figure 8F-H).

### Validation of CRGs in HNSCC

**Figure 9:**
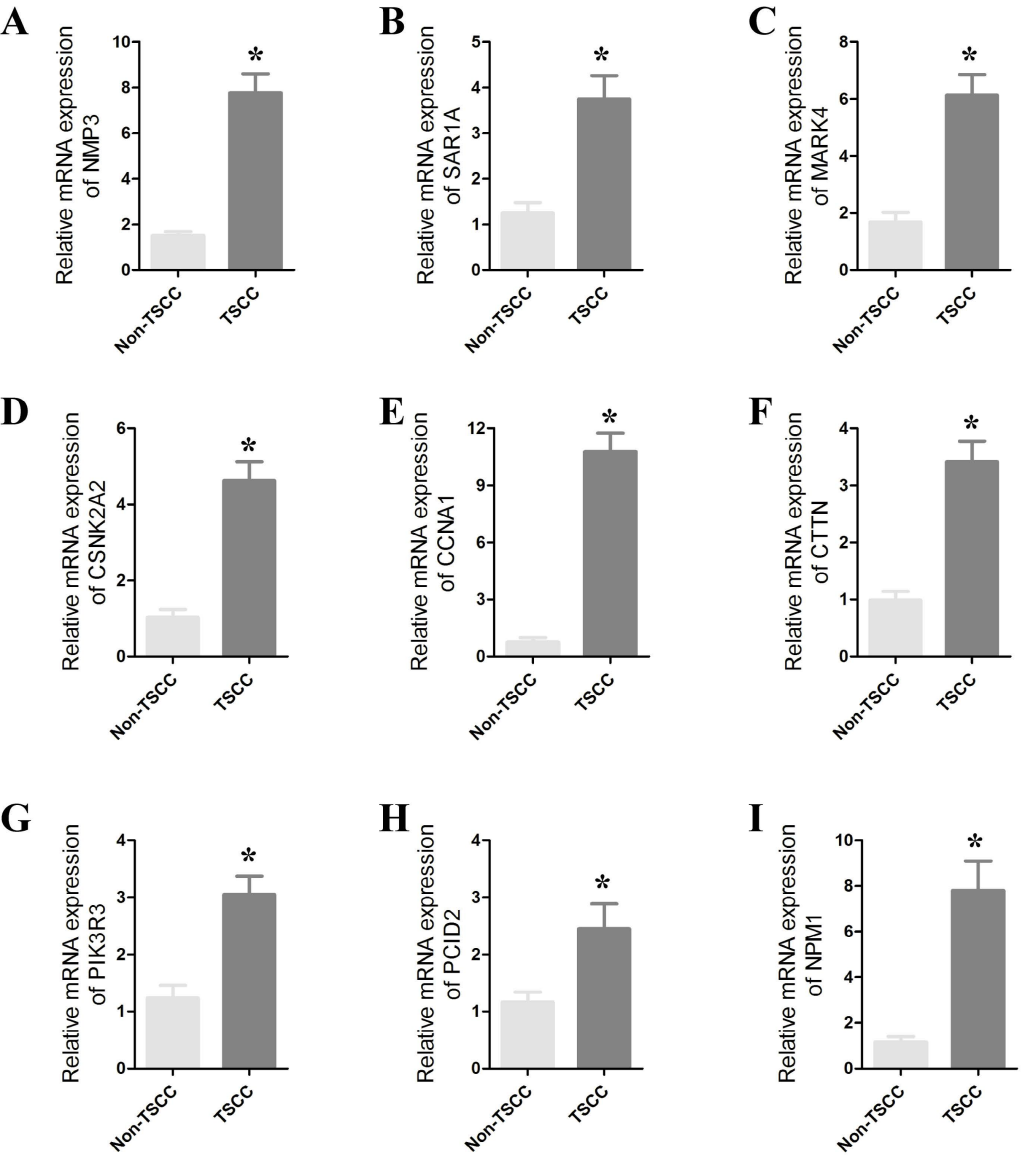
(A-H) qRT-PCR results showing significant differences in the expression of SAR1A, NPM1, NMP3, CTTN, CSNK2A2, PIK3R3, PCID2, MARK4 and CCNA1 between cancers and paracancers. The data represent the mean ± SEM of at least three independent experiments. * p<0.05, versus Non-TSCC.

To confirm the role of CRGs in HNSCC, we further validated their differential expression in normal and cancer samples through qPCR experiments. qPCR was performed on 8 paired cancer and adjacent normal tissues to detect mRNA expression levels of the prognostic CRGs. We observed significant differences in the expression of SAR1A, NPM1, NMP3, CTTN, CSNK2A2, PIK3R3, PCID2, MARK4 and CCNA1 between cancer and normal tissues (Figure 9 and Figure S4). The expression of these genes was significantly upregulated in cancer tissues. These results strongly support the reliability of our bioinformatics analysis.

## Discussion

Despite advancements in treatment modalities, the 5-year overall survival rate for Head and Neck Squamous Cell Carcinoma (HNSCC) patients remains below 50%. Due to the lack of effective early monitoring and screening factors, early detection is challenging. By the time HNSCC is diagnosed, it is often in the advanced stages with a dismal prognosis. Therefore, identifying ideal biomarkers for predicting HNSCC metastasis and prognosis is crucial. However, risk stratification based solely on tumor size, lymph node, and distant metastasis, as well as histological grading, is insufficient to predict the prognosis of HNSCC patients. There is an urgent need for more accurate prognostic models. The impact of centrosomes on tumors has been studied previously, with multiple genes identified as regulators of centrosomes playing key roles in HNSCC. In this study, we systematically identified the expression of CRGs in HNSCC and selected 11 genes associated with prognosis to construct a survival prediction model for HNSCC patients. The AUC values for the training and test groups were both greater than 0.5. Compared to other clinical factors, the centrosome-related prognostic model demonstrated higher prognostic value.

Validation using TCGA and an independent dataset (GSE41613) indicated that the predictive model could be a more accurate prognostic indicator for patient outcomes. The model stratified HNSCC patients into high-risk and low-risk groups, with the expected poorer overall survival in the high-risk group. Combining risk features with clinical information, we constructed more accurate nomograms to predict HNSCC patient overall survival. These findings collectively suggest that centrosome-related features serve as a reliable prognostic model for HNSCC.

Additionally, we analyzed the correlation between the prognostic features of each HNSCC patient and the immune landscape. The results indicated a significant association between risk scores and immune cell infiltration. The high-risk group showed a higher abundance of resting NK cells. Significant differences in CD8+ T cell infiltration were observed between different risk groups. Furthermore, patients in the high-risk group exhibited higher Tumor Mutational Burden (TMB) scores. Finally, we evaluated the predictive efficacy of the model for immunotherapy. Collectively, our findings suggest that our model may reflect immune infiltration and predict the response to immunotherapy in HNSCC.

Alterations in the structure, number, and function of centrosomes in cancer cells have been extensively documented. Numerous studies have demonstrated that centrosomes and their associated genes play a crucial role in tumor progression, and targeting centrosomes as a novel approach to cancer treatment has garnered significant attention. Abnormalities in centrosome number and structure are detected in nearly all human cancers^30^. Prognostic models about centrosomes have been applied in a variety of cancers to predict the prognostic characteristics of tumors. In previous studies, centrosome-associated gene signatures have been applied in a variety of tumors such as breast cancer, hepatocellular carcinoma, melanoma and low-grade glioma. And the prognostic model constructed based on centrosome genes predicted clinical information such as OS in tumor patients. GO, KEGG enrichment analysis of this model predicted its role in tumor progression. In addition, the model was further used to predict the level of mutational landscape, degree of immune cell infiltration and immune checkpoint expression in tumor patients. Based on this, a series of anti-cancer drug candidates with high sensitivity to tumor patients were screened^31^-^34^. In a study on breast cancer, centrosome amplification was found to trigger cell invasion, a behavior similar to that induced by the overexpression of the breast cancer oncogene ErbB2, and it further enhanced ErbB2-induced invasiveness. The researchers discovered that by increasing centrosomal microtubule nucleation, centrosome amplification activates Rac1, which disrupts normal cell-to-cell adhesion and promotes invasion^35^. Additionally, centrosome amplification has been associated with, and is sufficient to promote, the secretion of small extracellular vesicles (SEVs) in pancreatic cancer cells. In cancer, altered secretion of extracellular vesicles (EVs) contributes to tumor growth and metastasis^36^. Therefore, understanding the pivotal role of centrosomes and utilizing centrosome-related prognostic markers could facilitate the development of personalized cancer therapies. It was previously observed that the number and structural abnormalities of centrosomes differ significantly between normal squamous epithelial cells and tumor cells (both p<0.0001), suggesting that centrosome abnorm ies may play a critical role in tumor progression in head and neck squamous cell carcinoma (HNSCC)^37^ . Additionally, some studies have found that centrosome hyper-amplification occurs at a very high frequency in HNSCC and have explored its potential as a marker for tumor recurre.

Centrosome aberrations are closely linked to the tumor immune microenvironment (TIME). Therefore, we focused on investigating the differences in immune cell subpopulation composition between different risk groups. TIME comprises tumor cells, immune cells, and cytokines, and the interactions between these components, categorized as either anti-tumor or pro-tumor, determine the direction of anti-tumor immunity^38^. Our findings revealed that cancer-associated fibroblasts (CAF) and myeloid-derived suppressor cells (MDSCs) were highly expressed in the high-risk group, while CD8+ T cells were significantly elevated in the low-risk group. CAFs, also known as activated fibroblasts, are central components of the reactive stroma within TIME. CAFs are activated through diverse pathways and contribute to tumor growth, angiogenesis, invasion, metastasis, extracellular matrix (ECM) remodeling, and even chemotherapy resistance. CAFs interact with other immune components within TIME, establishing an immunosuppressive microenvironment that enables cancer cells to evade immune surveillance^39^. CAFs primarily remodel the ECM, creating a physical barrier between immune cells and cancer cells, thereby preventing immune cell infiltration and migration, ultimately suppressing the immune response to tumors. There is a strong correlation between ECM remodeling and cancer chemotherapy resistance, as it can prevent T cell attacks by therapeutic PD-1 inhibitors, thus promoting resistance to immune checkpoint inhibitors^40^. MDSCs, derived from hematopoietic stem cells (HSCs), play a pivotal role in promoting tumor immune escape by inhibiting tumor-killing immune cells and acting synergistically with other suppressive immune cells. Tumor-infiltrating MDSCs typically express high levels of PD-L1, and they increase PD-L1 expression by interacting with PD-1 on T cells, leading to T cell dysfunction^41^. MDSCs can also induce T cell autophagy, cell cycle arrest, and even cell death by depleting essential amino acids necessary for T cell growth and differentiation^42^. In our centrosome-associated prognostic model, patients in the high-risk group exhibited an inhibitory immune microenvironment with higher infiltration of CAFs and MDSCs, suggesting that these patients are in an immunosuppressive state. This finding is consistent with the survival curve predictions from our model, which may explain the poor prognosis observed in the high-risk group. Thus, centrosome aberrations may be able to remodel tumor TIME to promote immune escape of tumor cells.

To facilitate the clinical application of CRGs, candidate genes for the centrosome-related prognostic model were determined using univariate Cox regression analysis and the LASSO algorithm, resulting in the identification of YPEL1, NPM1, CSNK2A2, CCNA1, CTTN, PCID2, PIK3R3, MARK4, SAR1, NMP3, and CCND1. YPEL1, located on human chromosome 22q11.2, is reported to be a nuclear protein with potential regulatory roles in cell division^43^. In various cancer types, YPEL1 may exhibit either oncogenic or anti-tumor functions^44^. NPM1 is a multifunctional protein crucial for cell cycle control and centrosome replication^45^. Its overexpression has been reported in various tumors, promoting tumorigenesis and progression^46^. CSNK2A2, a serine/threonine protein kinase, is involved in cell cycle control and apoptosis^47^. CCNA1 regulates DNA synthesis and replication, playing a vital role in the transition from the G1 phase to the S phase of the cell cycle^48^. CTTN, a cytoskeleton-related scaffold protein, promotes cancer cell invasiveness in many tumors^49^. PCID2, also known as CSN12, has been studied for its molecular mechanisms in colorectal cancer^50^. PIK3R3, part of the PI3K regulatory domain, is upregulated in various cancers, playing a crucial role in tumorigenesis, cell proliferation, and metastasis^51^. MARK4, a member of the MARK family, is associated with diseases such as cancer, Alzheimer's, and metabolic syndrome^52^. SAR1, involved in regulating COPII vesicle assembly on the endoplasmic reticulum membrane, plays a role in tumor development^53^. NMP3, associated with diseases including lung adenocarcinoma, is known for its strong expression in various cell types, primarily localized in the cell nucleus. CCND1 is a key activator of cyclin-dependent kinase 4/6 in the cell cycle, critical for initiating DNA replication^54^.

Inevitably, this study has several limitations. Primarily, our model should be validated by additional datasets or prospective trials to enhance the model's generalizability and the persuasiveness of the study. Additionally, it is needed to further elucidate the mechanisms and functions of centrosome features in tumor development and HNSCC progression.

## Conclusions

In summary, based on 11 selected CRGs, we have established a novel prognostic model for HNSCC, which was externally validated. This model provides a new reference for predicting the prognosis, immune infiltration, and immunotherapeutic response of HNSCC. *In vitro* evaluation of centrosome-related gene expression further highlights the clinical significance of CRGs in HNSCC patients, offering new insights for developing more effective therapeutic targets in the future.

## Supplementary Material

Supplementary figures and table.
