# Supplementary material for: Prognostic model based on centrosome-related genes constructed in head and neck squamous cell carcinoma
Source: J Cancer. 2024 Oct 21;15(20):6531–44. doi: 10.7150/jca.102057 (PMC11632974; doi:10.7150/jca.102057)
Supplement: Supplementary file 1 — Supplementary figures and table. [file jcav15p6531s1.pdf]

Supplementary information

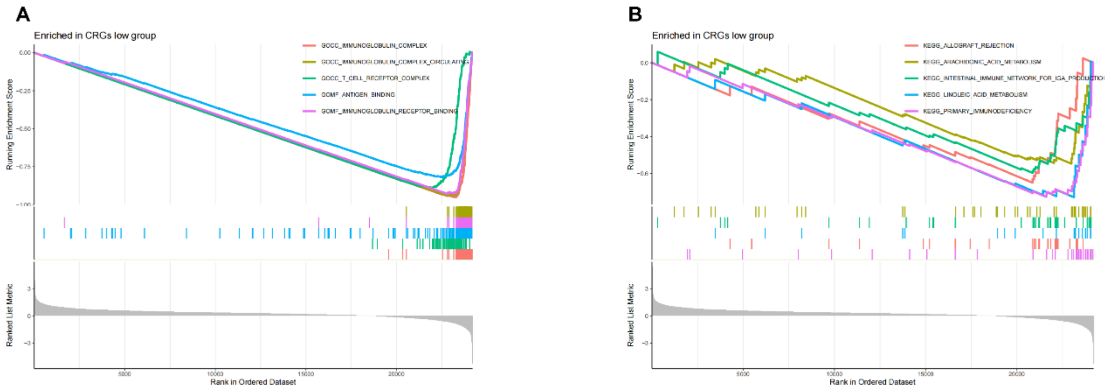

Figure S1. A and B, Genomic enrichment analysis (GSEA) of high/low expression groups of centrosome-related genes

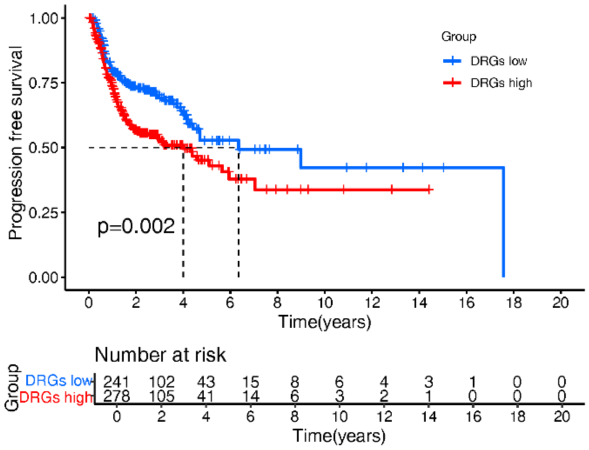

Figure S2. Kaplan-Meier analysis of progression free survival curves for TCGA patients in the DRGs high/low groups.

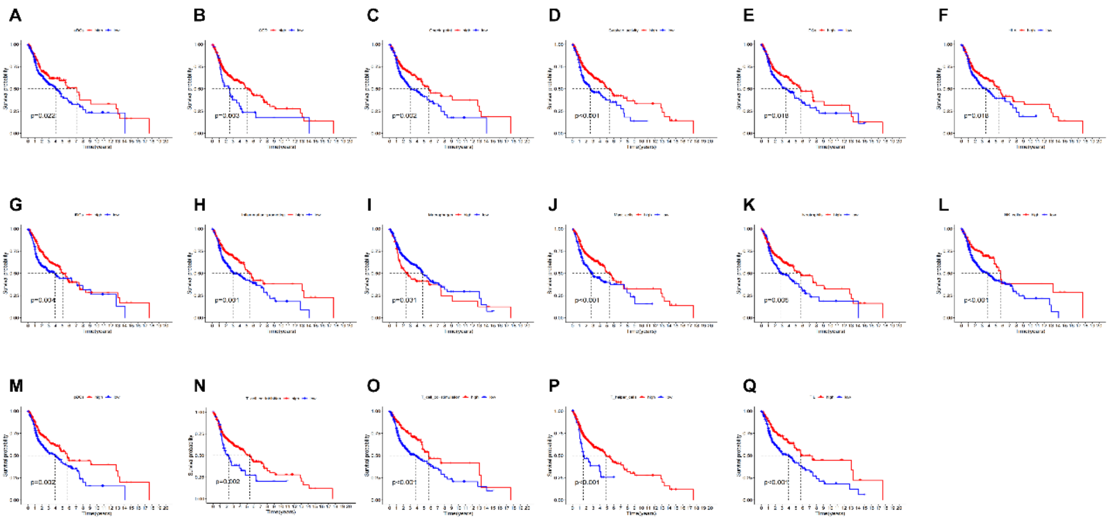

Figure S3. A to T, Kaplan-Meier analysis of OS curves in patients with high and low immunocompetence differences.

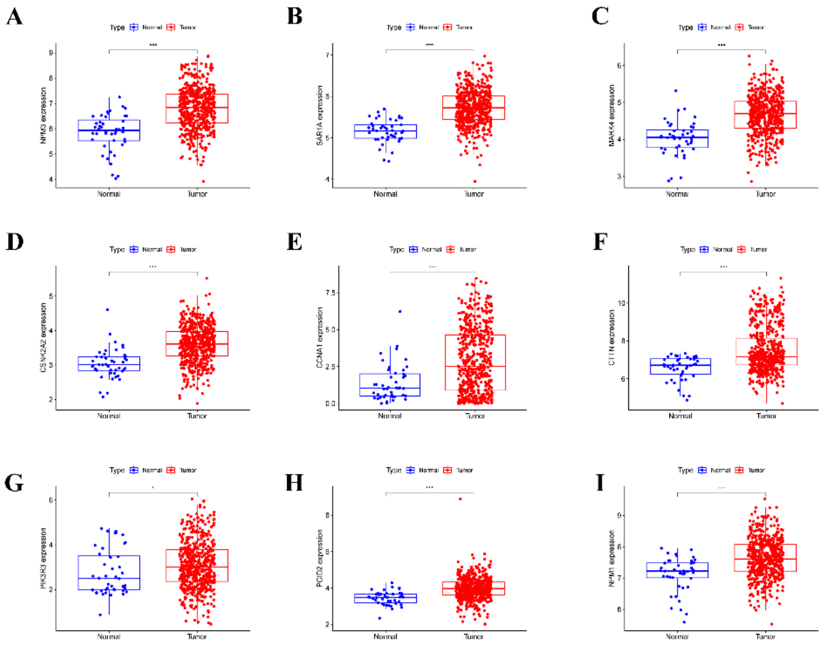

Figure S4. mRNA expression of SAR1A, NPM1, NMP3, CTTN, CSNK2A2, PIK3R3, PCID2, MARK4 and CCNA1 between normal and tumor in TCGA.

Table S1 Primer sequences used for Q-PCR and Real-time PCR

Gene Name	Primer	Sequence
CCNA1	Forward<br>Reverse	GCACACTCAAGTCAGACCTGCA<br>ATCACATCTGTGCCAAGACTGGA
PIK3R3	Forward<br>Reverse	CCACCTAAGCCAATGACTTCAGC<br>GTTGAGGCATCTCGGACCAAGA
CSNK2A2	Forward<br>Reverse	CGACCATCAACAGAGACTGACTG<br>GTGAGACCACTGGAAAGCACAG
CTTN	Forward<br>Reverse	AGGTGTCCTCTGCCTACCAGAA<br>CCTGCTCTTTCTCCTTAGCGAG
NMP3	Forward<br>Reverse	TGACCATCAGGAGATCGCAGTC<br>TTCAGGCGGAAGGTTACAGGTG
NPM1	Forward<br>Reverse	GCCAGTGCATATTAGTGGACAGC<br>GGAACCTTGCTACCACCTCCAG
PCID2	Forward<br>Reverse	CTGCGGAATCTTCCTCATCCTG<br>GGCAACCAGAAAAGCATCCAGAG
SAR1A	Forward<br>Reverse	GGGCTTTATGGACAGACCACAG<br>CCGTAACCTTGCCTCTTGAGCA
MARK4	Forward<br>Reverse	GTCAACAGACTGTGAGAGCATCC<br>GCTCTGTGTATGGCTTCAACTCC
ACTB	Forward<br>Reverse	CACCATTGGCAATGAGCGGTTC<br>AGGTCTTTGCGGATGTCCACGT
